# CD148 Tyrosine Phosphatase Promotes Cadherin Cell Adhesion

**DOI:** 10.1371/journal.pone.0112753

**Published:** 2014-11-11

**Authors:** Keiko Takahashi, Anton Matafonov, Katherine Sumarriva, Hideyuki Ito, Colette Lauhan, Dana Zemel, Nobuo Tsuboi, Jin Chen, Albert Reynolds, Takamune Takahashi

**Affiliations:** 1 Department of Medicine, Vanderbilt University School of Medicine, Nashville, TN, United States of America; 2 Department of Cancer Biology, Vanderbilt University School of Medicine, Nashville, TN, United States of America; Northwestern University Feinberg School of Medicine, United States of America

## Abstract

CD148 is a transmembrane tyrosine phosphatase that is expressed at cell junctions. Recent studies have shown that CD148 associates with the cadherin/catenin complex and p120 catenin (p120) may serve as a substrate. However, the role of CD148 in cadherin cell-cell adhesion remains unknown. Therefore, here we addressed this issue using a series of stable cells and cell-based assays. Wild-type (WT) and catalytically inactive (CS) CD148 were introduced to A431D (lacking classical cadherins), A431D/E-cadherin WT (expressing wild-type E-cadherin), and A431D/E-cadherin 764AAA (expressing p120-uncoupled E-cadherin mutant) cells. The effects of CD148 in cadherin adhesion were assessed by Ca^2+^ switch and cell aggregation assays. Phosphorylation of E-cadherin/catenin complex and Rho family GTPase activities were also examined. Although CD148 introduction did not alter the expression levels and complex formation of E-cadherin, p120, and β-catenin, CD148 WT, but not CS, promoted cadherin contacts and strengthened cell-cell adhesion in A431D/E-cadherin WT cells. This effect was accompanied by an increase in Rac1, but not RhoA and Cdc42, activity and largely diminished by Rac1 inhibition. Further, we demonstrate that CD148 reduces the tyrosine phosphorylation of p120 and β-catenin; causes the dephosphorylation of Y529 suppressive tyrosine residue in Src, a well-known CD148 site, increasing Src activity and enhancing the phosphorylation of Y228 (a Src kinase site) in p120, in E-cadherin contacts. Consistent with these findings, CD148 dephosphorylated both p120 and β-catenin *in vitro*. The shRNA-mediated CD148 knockdown in A431 cells showed opposite effects. CD148 showed no effects in A431D and A431D/E-cadherin 764AAA cells. In aggregate, these findings provide the first evidence that CD148 promotes E-cadherin adhesion by regulating Rac1 activity concomitant with modulation of p120, β-catenin, and Src tyrosine phosphorylation. This effect requires E-cadherin and p120 association.

## Introduction

Tyrosine phosphorylation, which is controlled by the balanced actions of protein tyrosine kinases (PTKs) and phosphatases (PTPs), plays a pivotal role in regulation of a variety of cellular signals, including proliferation, differentiation, migration, and adhesion. CD148 (also known as DEP-1, PTPη, PTPRJ) is a transmembrane PTP that is composed of an extracellular domain containing type III fibronectin repeats, a transmembrane segment, and a single intracellular catalytic domain [1]. CD148 is expressed in multiple cell types, including epithelial cells, endothelial cells, and hematopoietic lineages [2]–[4]. A body of evidence has shown a role for CD148 in negative regulation of cell proliferation and transformation. CD148 is down-regulated in cancer cell lines, correlated with their malignant phenotype [5], [6], and restoration of CD148 expression suppresses tumor growth in culture and *in vivo* [6]–[9]. Consistent with this finding, CD148 was shown to dephosphorylate and suppress growth factor receptors and their signaling proteins, including EGFR [10], [11], HGFR [12], [13], VEGFR2 [14], [15], Erk1/2 [11], [16], PLCγ1 [17], and p85 [18]. On the other hand, CD148 was also shown to dephosphorylate the suppressive tyrosine residue (Y529) in Src tyrosine kinase, increasing its activity, and support cell-matrix attachment [19] and Akt cell survival signals [15]. Collectively, these findings suggest that CD148 transduces cell static signals in adherent cells, arresting cell growth and migration and strengthening cell-matrix adhesion without reducing cell survival signals. In this context, it is of note that loss of heterozygosity at the ptprj (CD148) locus is frequently observed in human cancers [20].

CD148 was initially cloned from Hela cells as a PTP whose expression and activity is increased with cell density [1]. Further, several lines of evidence suggest a role for CD148 in regulation of cell-cell adhesion. First, CD148 is distributed to cell junctions as well as the cell surface [3], [21]. Second, CD148 associates with VE-cadherin in endothelial cells and its activity is increased with cell density [13], [22]. Third, the substrate trapping approach identified p120 catenin (p120) as a CD148 substrate [12], [21]. In aggregate, these findings suggest that CD148 interacts with the cadherin/catenin complex and regulates its function through p120. However, to date, the role of CD148 in cadherin cell adhesion remains unknown.

The cadherin/catenin complex is a major component of adherens junctions and plays a central role in cell-cell adhesion. The extracellular domain of cadherin mediates homophilic and calcium-dependent adhesion between adjacent cells and its cytoplasmic domain binds to the Armadillo-family proteins, p120 and β-catenin [23]. A body of literature has shown critical roles of p120 and β-catenin in cadherin cell-cell adhesion. p120 binds to the juxtamembrane domain of classical cadherins and stabilizes the cadherin complex at the cell surface [24], [25]. Importantly, p120 was shown to serve as a regulator of Rho- family small GTPases, which control actin cytoskeleton dynamics and play pivotal roles in the establishment of cell–cell contacts. Cadherin-bound p120 suppresses RhoA activity via recruitment of p190 Rho GTPase-activating-protein [26], [27]. p120 also interacts with, and recruits, ROCK1, a RhoA effector, to the cadherin complex [28], suggesting that p120 may function as a signaling scaffold to localize RhoA activity to adherens junctions. Further, several lines of evidence suggest that p120 regulates adhesive contact area by recruiting Rac1 to the cadherin complex [29], [30], although the detailed mechanism of this is currently unknown. On the other hand, β-catenin binds to the C-terminal tail of cadherin and mediates adhesive strength via α-catenin, which physically and functionally links cadherin to the actin cytoskeleton [31], [32]. Both p120 and β-catenin are known to be tyrosine phosphorylated [33], [34]. Indeed, p120 was identified as a highly tyrosine phosphorylated protein in v-Src transformed cells [35]. Although tyrosine phosphorylation of the cadherin complex has often been proposed as a mechanism that contributes to regulation or perturbation of cadherin function [36], detailed definition of the mechanisms that regulate tyrosine phosphorylation of the cadherin/catenin complex and its consequences for cadherin cell adhesion are currently incomplete.

In this study, we investigated the effects of CD148 in E-cadherin mediated cell-cell adhesion by introducing CD148 and E-cadherin forms into the cadherin-deficient A431D cells and by functionally and biochemically assessing E-cadherin mediated cell adhesion events in these cells. The p120-uncoupled E-cadherin mutant was also introduced to determine the contribution of p120 to CD148 effects. Our data demonstrate that CD148 promotes E-cadherin cell adhesion by regulating Rac1 activity, concomitant with modulation of p120, β-catenin, and Src tyrosine phosphorylation, and that this effect requires E-cadherin and p120 association.

## Materials and Methods

### Antibodies

The primary antibodies used for immunoblotting and immunoprecipitations: p120 (pp120), E-cadherin (34-E-cadherin), β-catenin, phospho-p120 (pY228), phosphotyrosine (pY20), Rac1, and Cdc42 were from BD Bioscience (San Jose, CA). β-actin (C-2), CD148 (143-41), and RhoA (119) were from Santa Cruz Biotechnology (Santa Cruz, CA). Src was from Upstate Biotechnology (Lake Placid, NY). Phospho-Src (pY529) was from Invitrogen Corporation (Carlsbad, CA). Secondary antibodies for immunoblotting: HRP-conjugated anti-mouse or anti-rabbit IgG were from GE Healthcare Bio-Sciences (Pittsburgh, PA). For immunoprecipitation of E-cadherin, anti-E-cadherin (HECD-1) from Takara Bio Company (Madison, WI) was used. For flow cytometry, phycoerythrin (PE)-conjugated anti-CD148 (143-41) from R&D Systems (Minneapolis, MN) was used. Immunofluorescence staining was performed using anti-E-cadherin (HECD-1, Takara Bio), anti-p120 (pp120, BD Bioscience), and the following secondary antibodies; FITC-conjugated anti-CD148 (143-41, Santa Cruz Biotechnology), Alexa Fluor 546-conjugated goat anti-mouse IgG2a (Invitrogen Corporation, Carlsbad, CA), and Alexa Fluor 647-conjugated goat anti-mouse IgG1 (Invitrogen Corporation).

### Cell Culture and Stable Cell Preparation

A431D epidermoid cervical carcinoma cells and its stable cells expressing either wild-type (WT) or 764AAA E-cadherin [25] were provided by Dr. Albert Reynolds (Vanderbilt University). A431 cells were purchased from American Type Culture Collection (Manassas, VA). These cells were cultured in DMEM (GIBCO Life Technologies, Grand Island, NY) supplemented with 10% FBS (Sigma Aldrich, St. Louis, MO), 1% L-glutamine, and 100 U/ml penicillin and 100 µg/ml streptomycin (GIBCO Life Technologies, Grand Island NY). CD148 stable cells were generated using the LZRS-IRES-Zeo retroviral vector as described [37]. Briefly, HA-tagged CD148 (WT or CS) cDNA sequences [18] were subcloned into the BamHI-EcoRI sites of pLZRS-IRES-zeo vector (provided by Dr. Al Reynolds, Vanderbilt University). Retrovirus was produced using Phoenix packaging cells [37]. Stable cell lines were selected with 400 µg/mL Zeocin (Invitrogen, Carlsbad, CA), stained with a PE-conjugated CD148 antibody (R&D Systems, Minneapolis, MN), and the cells whose CD148 levels are comparable to those in cultured human renal microvascular endothelial cells [38] were sorted using a BD FACSAria II flow cytometer (BD Biosciences, San Jose, CA). CD148-negative cells were also sorted and used as a control.

### Immunofluorescence Staining

Cells were plated onto glass coverslips (Fisher Scientific, Suwanee, GA) placed in a 12-well plate, cultured for 24 h, fixed in 100% methanol at −20°C for 7 min, and incubated with primary antibodies for 1 h at RT. The immunoreaction was visualized by the subsequent incubation with secondary antibodies (30 min at RT). Coverslips were mounted on glass slides with Fluorogel (Electron Microscopy Sciences, Hatifield, PA) and photographed using Zeiss LSM 510 confocal microscopy.

### Calcium-Switch Assay

Calcium-switch assay was performed as described previously [28]. In brief, cells were plated in 60 mm dish with growth medium and cultured for overnight. Then, the cells were starved in growth medium containing 0.1% FBS for overnight and the medium was replaced with LCM (calcium-free DMEM medium, GIBCO Life Technologies, Grand Island, NY) for 4 h and 1.8 mM CaCl_2_ was added for the indicated time. For immunofluorescence, cells were plated onto glass coverslips (Fisher Scientific, Suwanee, GA) placed in 12-well plate, cultured for 24 h, starved, and calcium-switch was carried out as described above. Then, the cells were fixed in 100% methanol at −20°C for 7 min, immunostained, and photographed as described above.

### Hanging-Drop Cell Aggregation Assay

Cell aggregation assay was performed as described previously [25]. Briefly, cells were trypsinized with 0.25% Trypsin EDTA (GIBCO Life Technologies, Grand Island, NY), washed twice in PBS, and re-suspended at the density of 5×10^5^ cells/ml in DMEM supplemented with 1% FBS. 2.5×10^4^ cells (in 50 µl) were pipetted on the lid of a 24-well plate, inverted, allowed to aggregate for 12–14 h in a humid 5% CO_2_ incubator at 37°C. Cells were subjected to shear force by passing them through a standard 200-µl pipet tip 10 times and were photographed through a microscope (Nikon, DIAPHOT Melville, NY) with 10×phase-contrast objective and Amscope digital camera (Amscope, Irvine, CA). For Rac 1 inhibition, Rac 1 inhibitor NSC 23766 (Chemdea LLC, Ridgewood, NJ) or Vehicle was added to the cell suspension at the concentration of 75 µM prior to hanging drop. For the assessment of Rho-family GTPase activity, cells were harvested at 0, 2, and 4 h after hanging drop, lysed, and the activities of Rac1, RhoA, and Cdc42 were measured as described below.

### RhoA, Rac1, and Cdc42 Activity Assay

RhoA, Rac1, and Cdc42 activities were assessed using the Rac1 and RhoA pull-down activation assay kit (Cytoskeleton, Inc., Denver, CO) according to the manufacturer’s instruction. In brief, cells were lysed in lysis buffer [50 mM Tris pH 7.5, 10 mM MgCl_2_, 0.5 mM NaCl, 2% NP-40, protease inhibitor cocktail (Cytoskeleton, Inc)] and the same amount (300–400 µg) of cell lysates was incubated with either 50 µg of PBD-GST or RBD-GST protein beads to precipitate activated Rac1 and Cdc42 or RhoA. Active (GTP-bound) and total Rac1, Cdc42, and RhoA were assessed by immunoblotting with anti-Rac1 (BD Bioscience), anti-Cdc42 (BD Bioscience), and anti-RhoA (Santa Cruz) antibodies. Relative levels of active versus total Rac1, Cdc42, and RhoA were quantified by densitometric analysis with image J (NIH) software. Experiments were repeated at least 4–5 times and the data was expressed as means ± S.E.M. ANOVA was used for multiple-group comparisons and the unpaired Student’s t test was used for two-group comparisons. Differences were considered statistically significant when *P*<0.05.

### Immunoprecipitation and Immunoblot Analysis

Immunoprecipitation and immunoblotting were performed as described previously [37], [38]. In brief, cells were washed with cold PBS and lysed in lysis buffer (20 mM HEPES/pH 7.5, 1% NP-40, 150 mM NaCl, 1 mM EDTA, 5 mM NaF, 5 mM iodoacetic acid, 1 mM Na_3_Vo_4_, protease inhibitor cocktail). The clarified cell lysates (20 µg) were separated by SDS-PAGE and immunoblotted with specific antibodies. For immunoprecipitation of E-cadherin complex or CD148, cells were lysed in CSK buffer (10 mM PIPES/pH 6.8, 100 mM NaCl, 300 mM sucrose, 3 mM MgCl_2_, and 0.5% NP-40) and the cell lysates were incubated with the specific antibodies overnight at 4°C and were subsequently incubated with Protein-G sepharose beads (GE health care, Piscataway, NJ) for 1 h at 4°C. Species-matched IgG was used as a control. The bound protein was separated by SDS-PAGE and immunoblotted with the specific antibodies. Immunoreactions were visualized using the ECL (enhanced chemiluminescence) detection system (GE health care, Piscataway, NJ). Relative levels of phosphorylated versus total protein were quantified by densitometric analysis with image J (NIH) software.

### shRNA-mediated CD148 Knockdown

A431 cells at 50% confluence were infected with a lentivirus (1×10^6^ infectious units) encoding CD148-targeting or scrambled shRNA (Sigma, St.Louis, MO) in the presence of 5 µg/mL polybrene (Santa Cruz Biotechnology, Santa Cruz, CA) as described previously [38]. At 72 h after infection, cells were used for the study.

### GST Pull-Down Study

GST-CD148 (WT, DA) fusion proteins were prepared as described previously [18]. GST pull-down and vanadate competition experiments were also carried out as described previously [18]. In brief, A431D/E-cadherin WT or A431D cells were treated with or without 0.1 mM pervanadate for 20 min, rinsed twice with PBS, and lysed in lysis buffer [20 mM Tris/pH 8.0, 200 mM NaCl, 1 mM EDTA, 0.5% NP-40, protease inhibitor cocktail (Roche Applied Science, Indianapolis, IN)]. The lysates were pre-cleared using GST-conjugated glutathione agarose beads (GE health care, Piscataway, NJ) and then incubated with GST or GST-CD148 proteins (20 µg) overnight at 4°C. The GST complexes were pulled-down with glutathione-agarose beads and subjected to immunoblotting. For the vanadate competition, 2 mM Na_3_VO_4_ was added to the cell lysates. GST fusion proteins were also pre-incubated with 2 mM Na_3_VO_4_ prior to addition.

### *In Vitro* Dephosphorylation Assay

*In vitro* dephosphorylation assay was performed as described previously [18], [21]. In brief, A431D/E-cadherin WT cells were treated with or without 0.1 mM pervanadate for 20 min, rinsed with PBS, and lysed in HNTG lysis buffer [50 mM HEPES/pH 7.5, 150 mM NaCl, 1 mM EGTA, 1.5 mM MgCl_2_, 10% glycerol, and 1% Triton X-100, 1 mM Na_3_VO_4_, protease inhibitor cocktail (Roche Applied Science, Indianapolis, IN)]. p120, β-catenin, and E-cadherin were immunoprecipitated from the lysates with specific antibodies. The immunoprecipitates were washed twice in wash buffer [50 mM HEPES/pH 7.5, 150 mM NaCl, 10% glycerol, 0.1% (v/v) Triton X-100, and 1 mM EDTA] and subsequently in succinate buffer [50 mM succinate/pH 6.0, 50 mM NaCl, 1 mM EDTA, and 1 mM dithiothreitol]. The beads were then suspended in 100 µl of succinate buffer with either GST or GST-CD148 proteins (WT, CS) and incubated for 30 min at 30°C. After washing with succinate buffer, the immunoprecipitates were subjected to immunoblotting. For the vanadate competition, 1 mM Na_3_VO_4_ was added to the reaction mixture prior to incubation.

## Results

### The effects of CD148 on the expression, complex formation, and junctional distribution of E-cadherin

**Figure 1:**
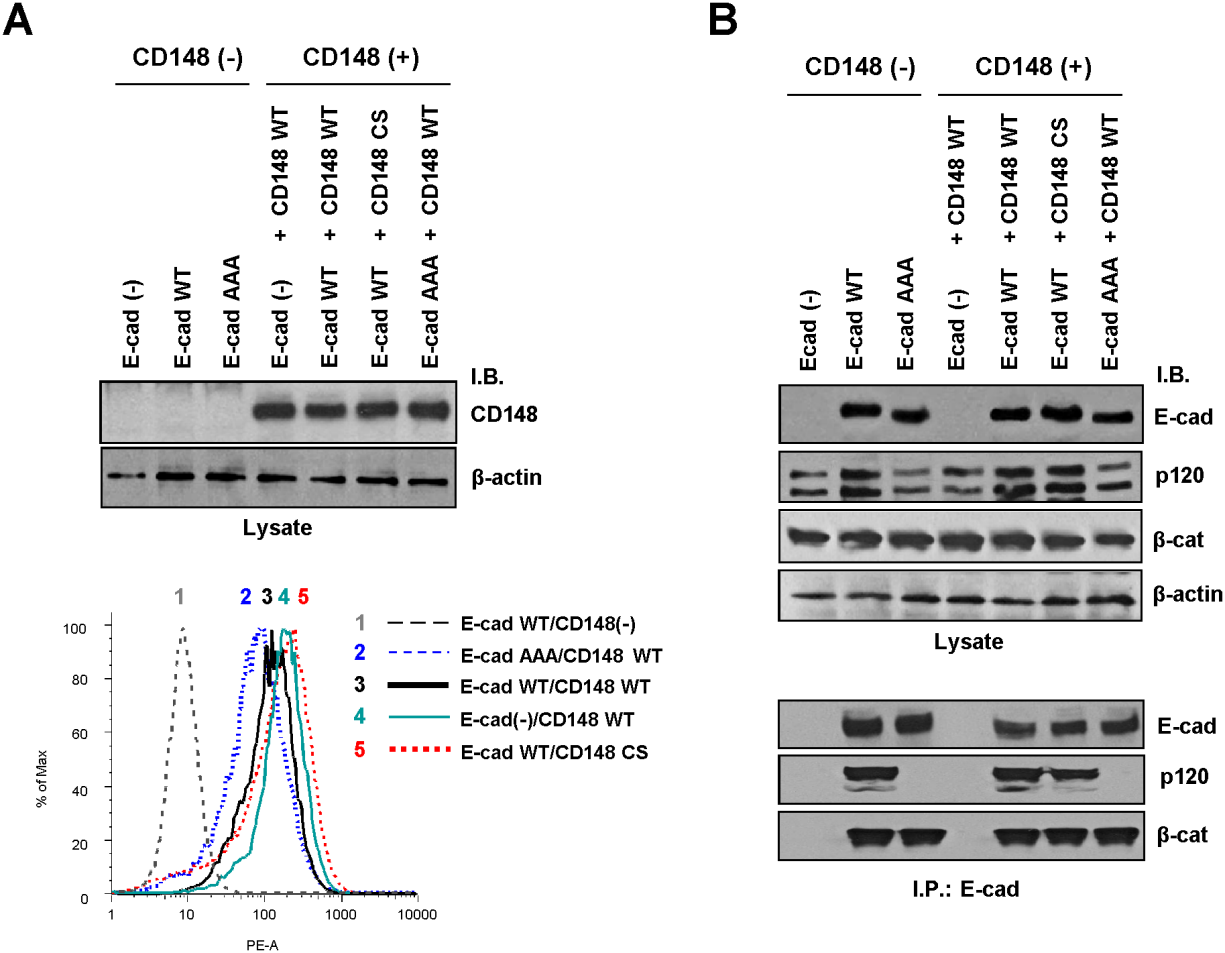
Introduction of CD148 forms to A431D and A431D/E-cadherin cells and its effects for E-cadherin and catenin expression and complex formation. **A)** Wild-type (WT) or catalytically inactive (CS) CD148 was stably introduced to A431D cells lacking classical cadherins [E-cad (−)] or expressing wild-type (WT) or p120-uncoupled mutant (764AAA) E-cadherin. The expression levels of CD148 in these cells were examined by immunoblotting (upper panel) and flow cytometry (lower panel). The loading was assessed by reblotting the membrane for β-actin. **B)** The levels of E-cadherin, p120, and β-catenin in nearly confluent CD148 stable cells were assessed by immunoblotting, comparing with CD148-negative cells (upper panels). The formation of E-cadherin/catenin complex was assessed by co-immunoprecipitation with E-cadherin (lower panels). Note: The association of E-cadherin with p120 is not observed in A431D/E-cadherin 764 AAA cells.

**Figure 2:**
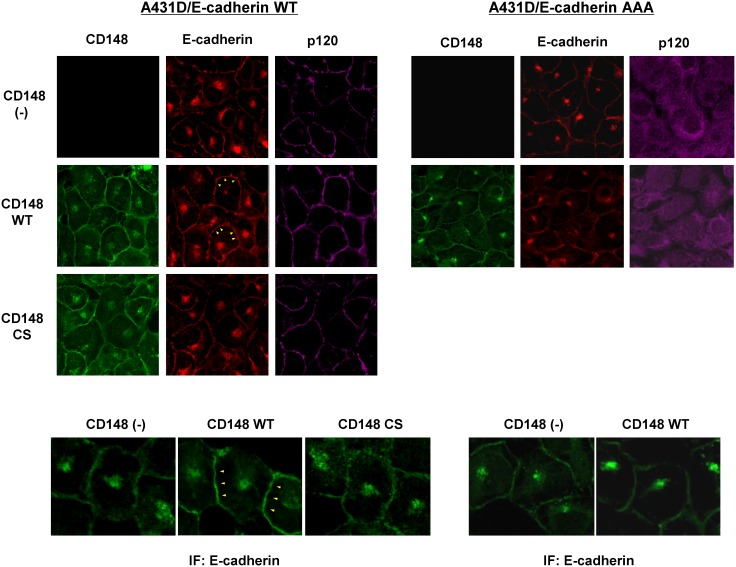
Effects of CD148 in E-cadherin distribution. Immunofluorescence localization of CD148 (green), E-cadherin (red), and p120 (purple) were examined in CD148 WT or CS-introduced A431D/E-cadherin WT (left panels) and A431D/E-cadherin 764AAA (right panels) cells and compared with CD148-negative cells. Lower panels show a higher magnification of E-cadherin immunofluorescence. Wild-type E-cadherin is more broadly distributed at cell junctions in CD148 WT-introduced cells (arrowheads in left panels), while the distribution of p120-uncoupled E-cadherin is unaltered by CD148 WT introduction (right panels).

CD148 is abundantly expressed in epithelial cells of various tissues [2]. E-cadherin, in general, plays a major role in cell-cell adhesion in this cell type. We therefore investigated the effects of CD148 on E-cadherin cell adhesion. For this, we utilized an experimental system of A431D cells. A431D cells lack the expression of classical cadherins [39]; therefore, introduction of E-cadherin allows the specific investigation of E-cadherin function. This experimental system was successfully applied to the structural and functional investigation of E-cadherin [25], [28]. Wild-type (WT) or catalytically inactive (C1239S, CS) forms [18] of CD148 were introduced into A431D or A431D/E-cadherin WT cells [25] in which wild-type E-cadherin is stably introduced. Since p120 was suggested to serve as a substrate for CD148, we also introduced CD148 into A431D/E-cadherin 764AAA cells [25] that express the p120-uncoupled E-cadherin mutant to determine the role of p120 in CD148 effects. Because excessive CD148 expression may induce non-physiological effects, the cells that express CD148 at levels comparable to those in cultured endothelial cells were sorted by flow cytometry and used in the study (**Figure S1**). Shown in **Figure 1A**, we confirmed the comparable levels of CD148 expression in the prepared stable cells by immunoblotting and flow-cytometric analysis. Using these cells, we first examined the effects of CD148 for the expression of E-cadherin and catenins and the formation of E-cadherin/catenin complex by immunoblot analysis and co-immunoprecipitation. Shown in **Figure 1B**, the cellular expression levels of E-cadherin, p120, and β-catenin (upper panels) and the E-cadherin and p120 or β-catenin associations (lower panels) were not altered by CD148 introduction in A431D/E-cadherin WT and A431D/E-cadherin 764AAA cells. As expected, E-cadherin and p120 association was not observed in A431D/E-cadherin 764AAA cells. The surface E-cadherin expression assessed by flow cytometry was also unaltered in CD148-introduced cells (data not shown). Therefore, we next assessed the cellular distribution of E-cadherin in CD148-introduced cells, compared with CD148-negative cells. Shown in **Figure 2** (left panels), E-cadherin was more broadly and intensely immunostained at cell junctions in CD148 WT, but not CS, introduced A431D/E-cadherin WT cells, while CD148 did not alter the E-cadherin distribution in A431D/E-cadherin 764AAA cells (right panels). p120 and β-catenin (data not shown) were co-localized with E-cadherin in A431D/E-cadherin WT cells, while p120 was distributed to the cytoplasm in A431D/E-cadherin 764AAA cells as described previously [25]. These findings demonstrate that CD148 WT, but not CS, promotes E-cadherin cell-cell adhesion and that this effect requires the E-cadherin and p120 association. It is of note that a similar effect was also observed with VE-cadherin distribution in CD148-overexpressed endothelial cells (**Figure S2**).

### CD148 promotes E-cadherin contact formation concomitant with an increase in Rac1 activity

**Figure 3:**
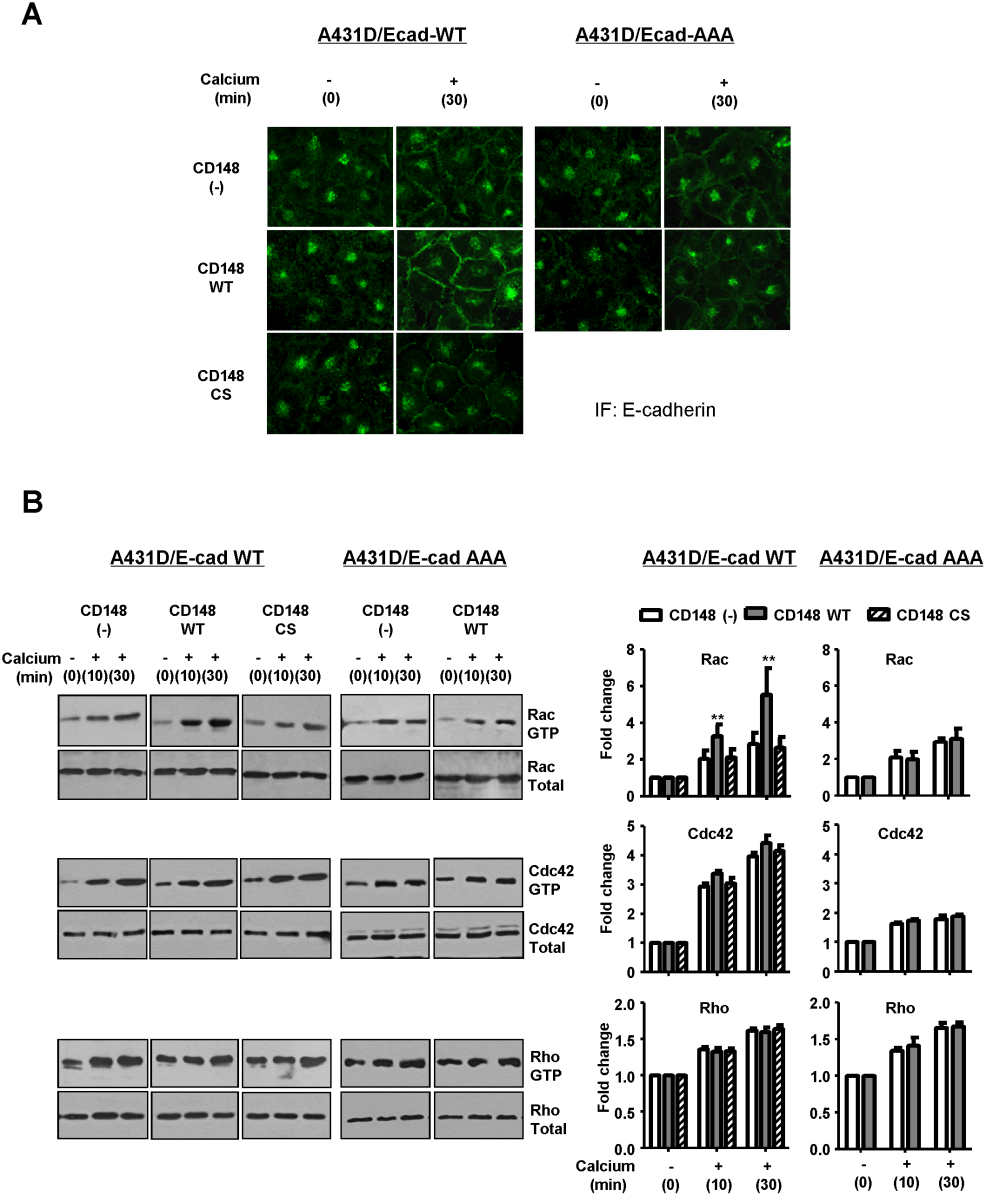
CD148 promotes E-cadherin contact formation with an increase in Rac1 activity. **A)** Effects of CD148 in E-cadherin contact formation were examined by a calcium-switch assay and immunofluorescence staining. CD148 WT, but not CS, promotes E-cadherin contact formation in A431D/E-cadherin WT cells, as evidenced by the expanded and intense E-cadherin distribution (left panels), while CD148 WT shows no effects in A431D/E-cadherin 764 AAA cells (right panels). **B)** Activities of Rac1, Cdc42, and RhoA were measured in the condition of calcium switch assay. Active and total levels of Rac1, Cdc42, and RhoA proteins were assessed by the pull-down assays and/or immunoblotting described in the “Materials and Methods” (left panels). The activity was normalized to total amount of protein using densitometry and expressed as fold change (right panels). The data show means ± SEM of quadruplicate determinations. **P<0.05 vs. CD148 (−) cells.

**Figure 4:**
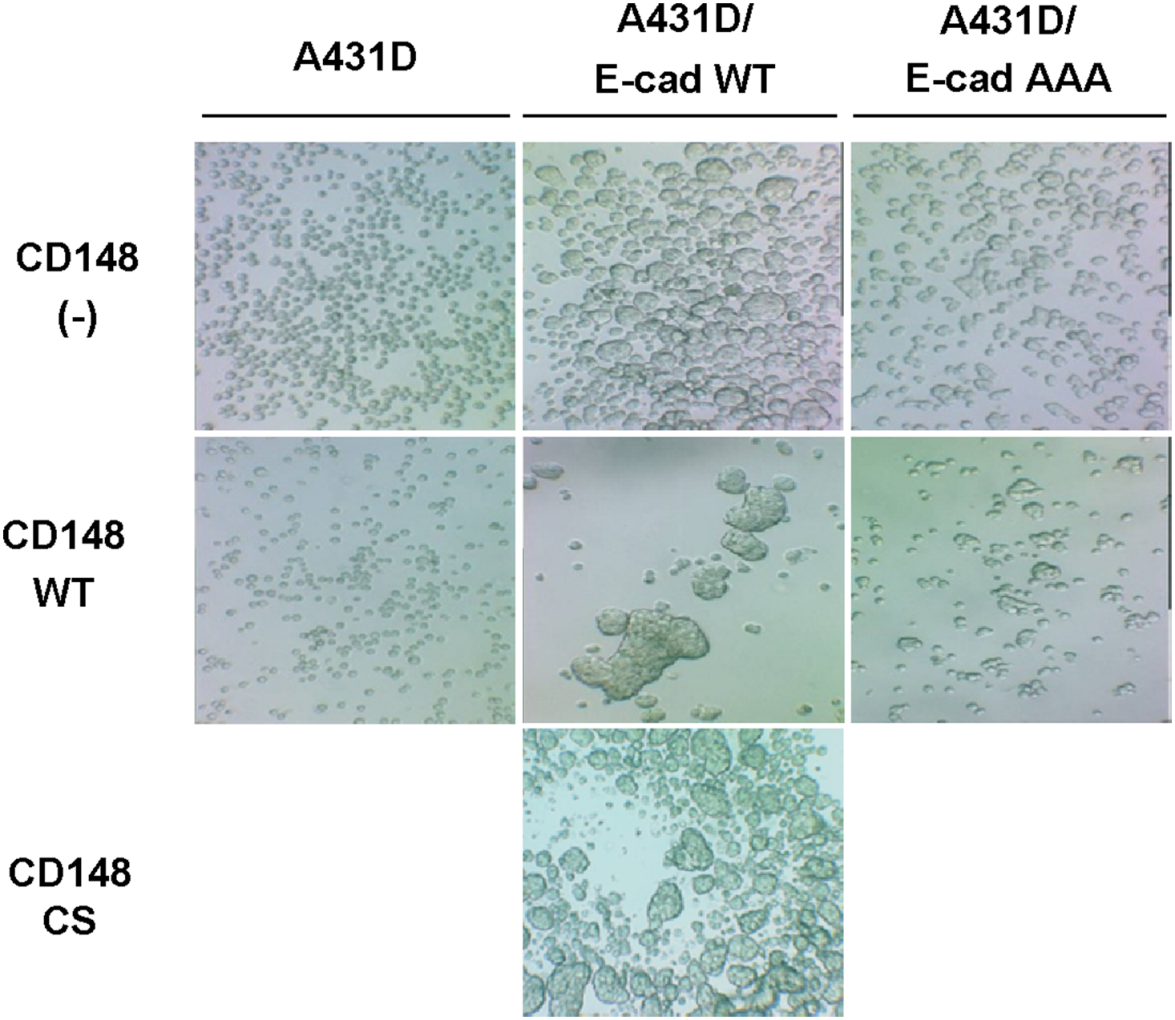
CD148 strengthens cell-cell adhesion in A431D/E-cadherin WT, but not A431D or A431D/E-cadherin 764AAA, cells. Effects of CD148 in cell-cell adhesion were assessed by a hanging drop assay. Images show representative data of ten independent experiments. CD148 WT, but not CS, remarkably increases the cell-cell adhesion in A431D/E-cadherin WT cells, while it shows no effects in A431D or A431D/E-cadherin 764 AAA cells.

**Figure 5:**
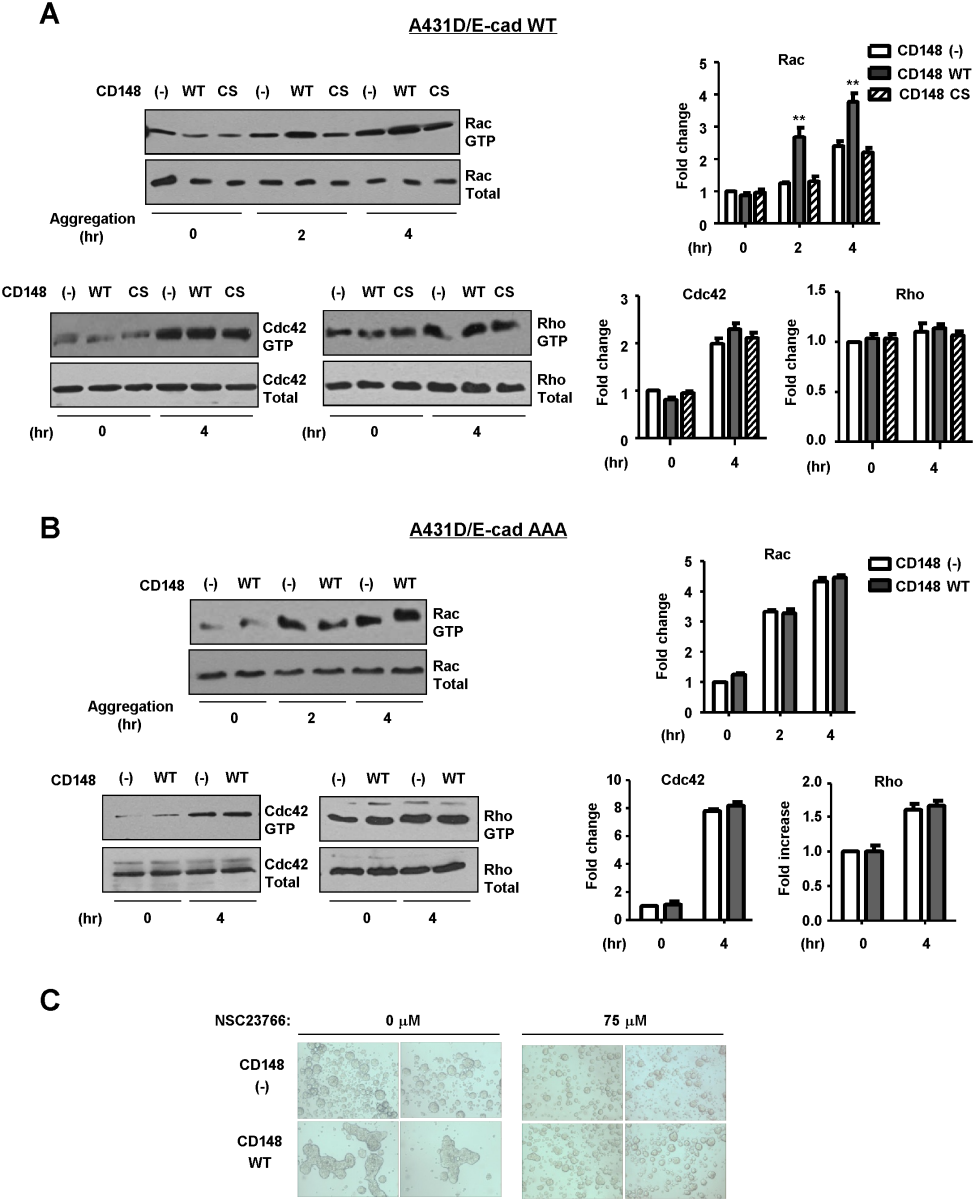
CD148 increases Rac1 activity in the condition of a hanging drop assay. **A and B)** CD148-introduced or CD148-negative A431D/E-cadherin WT (panel A) and A431D/E-cadherin 764 AAA (panel B) cells were subjected to a hanging drop assay. Rac1, Cdc42, and RhoA activities were assessed at the indicated time points. Active and total levels of Rac1, Cdc42, and RhoA proteins were assessed by pull-down assays and/or immunoblot analysis (left panels). The relative levels of active versus total Rac1, Cdc42, and RhoA were quantified by densitometric analysis (right panels). The data show means ± SEM of quadruplicate determinations. **P<0.05 vs. CD148 (−) cells. CD148 WT, but not CS, increases Rac1 activity in A431D/E-cadherin WT cells, while this effect is not observed in A431D/E-cadherin 764 AAA cells. **C)** Effects of CD148 WT in cell-cell adhesion were assessed by a hanging drop assay in the presence or absence of Rac1 inhibitor NSC 23766 (75 µM). CD148 increase of cell-cell adhesion is largely diminished by Rac1 inhibition.

**Figure 6:**
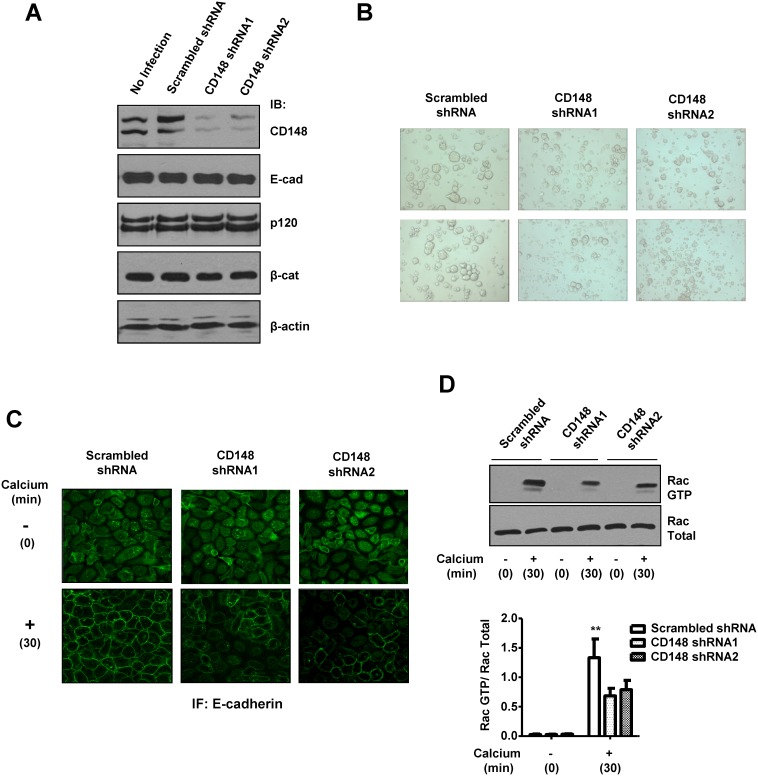
CD148 knockdown reduces cell-cell adhesion and E-cadherin contacts accompanied by a decrease in Rac1 activity in A431 cells. **A)** A431 cells was infected with a lentivirus encoding CD148-targeting or scrambled shRNA. Cells were harvested at 72 h after infection and the expression of CD148, E-cadherin, p120, and β- catenin was assessed by immunoblot analysis. Equal loading was confirmed by reblotting the membrane for β-actin. The targeting shRNA reduces CD148 expression (∼65%) without altering the E-cadherin, p120, and β-catenin expression. **B)** CD148 knock-down cells and the control cells treated with scrambled shRNA were subjected to a handing drop assay. Data are representative of five independent experiments. CD148 knockdown reduces cell aggregation in A431 cells. **C and D)** CD148 knock-down and control cells were subjected to a calcium switch assay and were immunostained for E-cadherin (panel C). Rac1 activity in these cells was also assessed (panel D). Representative results of four independent experiments are shown. The data show means ± SEM of quadruplicate determinations. **P<0.05 vs. CD148-targeting cells. CD148 knockdown reduces the E-cadherin contact formation accompanied by a decrease in Rac1 activity.

To validate the CD148 activity to promote E-cadherin cell-cell adhesion, we conducted two cell-based assays. First, we assessed the effects of CD148 in E-cadherin contact formation by a calcium switch assay. Since the Rho-family small GTPases, which control actin cytoskeleton dynamics, were shown to play critical roles in the establishment of E-cadherin cell-cell adhesion [40], the activities of Rac1, RhoA, and Cdc42 were also assessed by PBD-GST or RBD-GST pull-down assays. Shown in **Figure 3A**, CD148 WT, but not CS, promoted the formation of E-cadherin contacts after calcium addition in A431D/E-cadherin WT cells, as evidenced by expanded and intense E-cadherin immunoreactivity at cell junctions (left panels), while this effect was not observed in A431D/E-cadherin 764AAA cells (right panels). Further, the activity assays of Rac1, RhoA, and Cdc42 small GTPases showed that this CD148 WT effect is accompanied by an increase in Rac1, but not RhoA or Cdc42, activity (**Figure 3B**) and this activity was abolished by an E-cadherin neutralizing antibody (**Figure S3**), indicating that this effect is associated with E-cadherin cell adhesion. The CD148 WT induced increase of Rac1 activity was not observed with CD148 CS or in A431D/E-cadherin 764AAA cells. Second, we also addressed this issue using a hanging-drop cell aggregation assay as this assay was successfully applied to the assessment of E-cadherin function [25]. As shown in **Figure 4**, CD148 WT, but not CS, remarkably increased cell-cell aggregation in A431D/E-cadherin WT cells, while this effect was not observed in A431D or A431D/E-cadherin 764AAA cells. Further, shown in **Figure 5**, we also examined Rac1, RhoA, and Cdc42 activities in this condition and found that CD148 WT, but not CS, increases Rac1, but not RhoA or Cdc42, activity in A431D/E-cadherin WT cells (panel A), while this effect was not observed in A431D/E-cadherin 764AAA (panel B) and A431D (**Figure S4**) cells. Consistent with this finding, a Rac1 inhibitor (NSC23766) largely abolished the ability of CD148 WT to promote cell aggregation (panel C). These results were also supported by our cell density experiments showing that CD148 WT, but not CS, increases the Rac1 activity in a cell-density dependent manner in A431D/E-cadherin WT, but not 764AAA, cells (data not shown). Last, we asked if CD148 knockdown shows opposite effects on E-cadherin cell adhesion. For this experiment, we utilized A431 cells that endogenously express E-cadherin and CD148 (**Figure 6A**). CD148 was knocked-down using the lentivirus encoding CD148-targeted shRNA. Scrambled shRNA was used as a control. Shown in **Figure 6A**, CD148-targeted, but not scrambled, shRNA down-regulated the CD148 expression (∼65%) in A431 cells without altering the E-cadherin, p120, and β-catenin expression. Further, CD148 knock-down decreased the cell aggregation of A431 cells (**Figure 6B**) and the E-cadherin contact formation and Rac1 activation in the condition of calcium-switch assay (**Figure 6C&D**). Collectively, these results demonstrate that CD148 promotes E-cadherin cell-cell adhesion concomitant with an increase in Rac1 activity and that this requires the E-cadherin and p120 association.

### The effects of CD148 on tyrosine phosphorylation of E-cadherin/catenin complex

**Figure 7:**
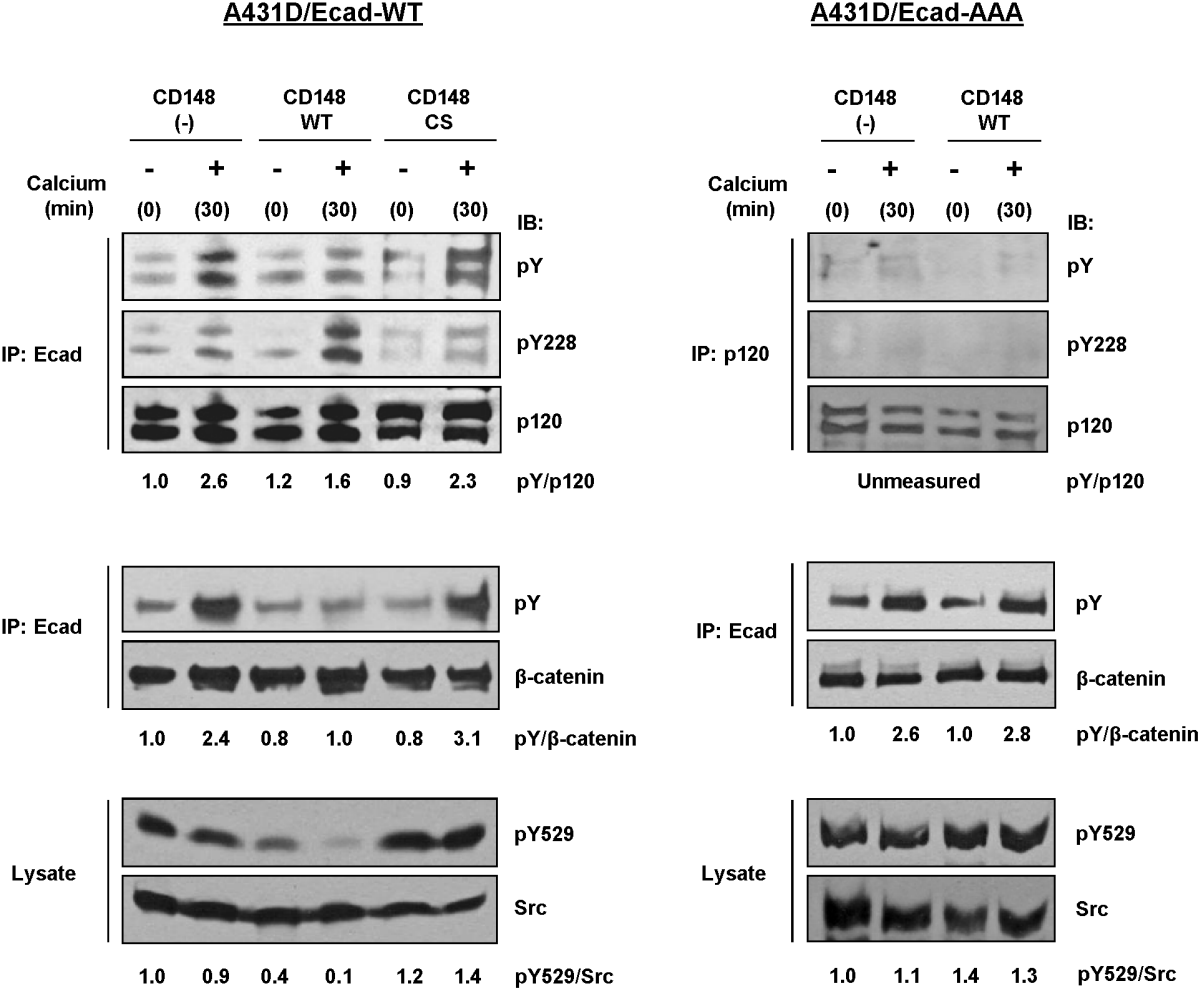
CD148 regulates the tyrosine phosphorylation of p120, β-catenin, and Src upon E-cadherin engagement. Effects of CD148 in the cadherin adhesion-associated tyrosine phosphorylation of p120, β-catenin, and Src were assessed by a calcium-switch assay and immunoblot analysis using A431D/E-caherin WT (left panels) and A431D/E-cadherin 764AAA (right panels) cells. For p120 and β-catenin, tyrosine phosphorylation of p120 and β-catenin that were co-immunoprecipitated with E-cadherin was assessed by immunoblotting. In A431D/E-cadherin 764 AAA cells, p120 was immunoprecipitated. The membranes were reprobed with p120, β-catenin and Src antibodies and a ratio of phosphorylated to total protein was quantified by densitometry. Data are representative of five independent experiments. CD148 WT, but not CS, reduces the tyrosine phosphorylation of p120, β-catenin, and Src (Y529) upon E-cadherin engagement in A431D/E-cadherin WT cells, while it increases the phosphorylation of Y228 (a Src site) in p120. These effects are not observed in A431D/E-cadherin 764 AAA cells.

To determine the biochemical changes in E-cadherin/catenin complex, which are associated with the CD148 effects, we next investigated the tyrosine phosphorylation of E-cadherin/catenin complex in the setting of calcium-switch assay. E-cadherin/catenin complex was immunoprecipitated using an E-cadherin antibody and the tyrosine phosphorylation of p120 and β-catenin that are recruited to E-cadherin was assessed by immunoblotting. Shown in **Figure 7** (left panels), CD148 WT, but not CS, reduced the tyrosine phosphorylation of p120 and β-catenin in E-cadherin contacts in A431D/E-cadherin WT cells, while this effect was not observed in A431D/E-cadherin 764AAA cells (right panels). In A431D/E-cadherin 764AAA cells, we examined the tyrosine phosphorylation of p120 that is present in cytoplasm by immunoprecipitation. Shown in right panels and **Figure S5**, tyrosine phosphorylation of p120 was highly limited in these cells compared to A431D/E-cadherin WT cells and CD148 WT showed no effects. It is well known that CD148 dephosphorylates the suppressive tyrosine residue (Y529) in Src tyrosine kinase and increases its activity [19], [41], [42]. Further, Src is known to play a key role in the establishment of E-cadherin cell-cell adhesion [43], [44]. Therefore, we also evaluated the dephosphorylation of Src Y529. Shown in **Figure 7**, CD148 WT, but not CS, dephosphorylated Src Y529 in E-cadherin contacts in A431D/E-cadherin WT cells (left panels), while this effect was not observed in A431D/E-cadherin 764AAA cells (right panels), suggesting that CD148 activity is increased in E-cadherin contacts in A431D/E-cadherin WT, but not 764AAA, cells. Consistent with this finding, CD148 WT increased the phosphorylation of p120 Y228 tyrosine residue (a Src kinase site) [34] in A431D/E-cadherin WT cells. The p120 present in the cytoplasmic fraction showed the limited tyrosine phosphorylation, including Y228, in A431D/E-cadherin WT cells as described in previous reports [25], [45], [46], and CD148 showed no effects (data not shown). In addition, we noted that CD148 WT increases the phosphorylation of Vav2 Y172 tyrosine residue (a Src site) [47] in A431D/E-cadherin WT cells (**Figure S6**). The β-catenin Y654 is also known to be phosphorylated by Src [48]; however, its phosphorylation was not increased by CD148 WT (data not shown). Although the mechanism of this is currently unknown, CD148 might dephosphorylate this tyrosine residue or increase the activity of the PTP that dephosphorylates this residue. E-cadherin tyrosine phosphorylation was highly limited in both A431D/E-cadherin WT and 764AAA cells, and CD148 showed no effects (data not shown). CD148 knock-down showed opposite effects for p120, β-catenin, and Src phosphorylation in A431 cells (data not shown), yet its effects were less evident, perhaps due to the low level expression of CD148 in this cells.

**Figure 8:**
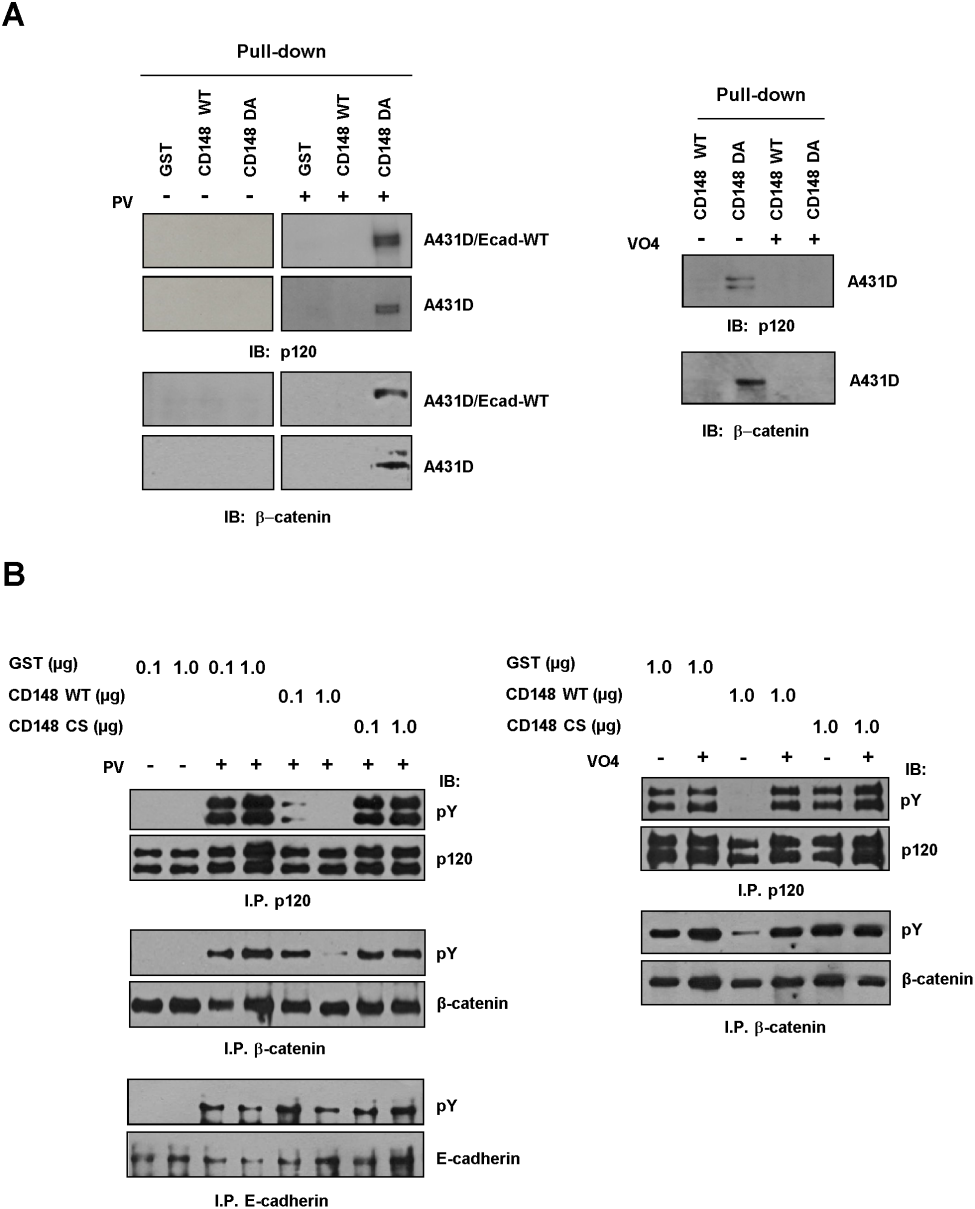
CD148 dephosphorylates p120 and β-catenin *in vitro*. **A)** A431D/E-cadherin WT and A431D cells were treated with (+) or without (−) pervandadate (PV) and cell lysates were incubated with GST or GST-CD148 (WT, DA) proteins. GST-protein complex were pulled-down using glutathione beads and the protein interactions were examined by immunoblotting (left panels). Vandadate (VO4) competition was also assessed (right panels). Substrate-trapping (DA), but not WT, form of CD148 binds to p120 and β-catenin in a phosphorylation dependent manner and these interactions are blocked by vanadate (VO4). **B)** CD148 dephosphorylation of E-cadherin, p120, and β-catenin was assessed *in vitro*. E-cadherin, p120, and β-catenin were immunoprecipitated from the pervanadate (PV)-treated or untreated A431D/E-cadherin WT cells. The immunoprecipitates were incubated with GST or GST-CD148 proteins and its effects were assessed by immunoblotting with a pY20 phosphotyrosine antibody (pY) (left panels). The amount of protein was assessed by reprobing the membranes with specific antibodies. Vanadate (VO4) competition was also assessed (right panels). CD148 WT, but not CS, dephosphorylates p120 and β-catenin in a dose dependent manner, while its effects for E-cadherin are limited. CD148 dephosphorylation of p120 and β-catenin is blocked by vanadate (VO_4_).

These results suggest that CD148 dephosphorylates p120 and β-catenin as well as Src. Therefore, we next addressed this issue using *in vitro* assays. First, we asked if the substrate trapping (D1205A, DA) form [18] of CD148 binds to the phosphorylated p120 or β-catenin. Tyrosine phosphorylation of p120 or β-catenin was induced by treating the cells with pervanadate. Shown in **Figure 8A** (left panels), the substrate trapping form of GST-CD148 (GST-CD148DA) protein strongly bound to the phosphorylated p120 and β-catenin in A431D/E-cadherin WT cells, while p120 and β- catenin binding to the wild-type CD148 (GST-CD148 WT) or control GST was limited. These protein interactions were also observed in A431D cells, indicating that they are not mediated by E-cadherin. Indeed, E-cadherin was not trapped by GST-CD148DA (data not shown). Further, the interaction of CD148 DA with phosphorylated p120 and β- catenin was blocked by the addition of vanadate, a PTP competitor, to the reaction (right panels). These findings suggest that both p120 and β-catenin serve as a substrate for CD148. Therefore, we next asked if CD148 dephosphorylates p120 and β-catenin *in vitro*. Since previous studies have shown that E-cadherin is phosphorylated by tyrosine kinases including Src [49], [50], we also examined CD148 dephosphorylation of E-cadherin, although its tyrosine phosphorylation was limited in intact cells. For this, p120, β-catenin, and E-cadherin were immunoprecipitated from the pervanadate-treated A431D/E-cadherin WT cells and reacted with different amounts of GST-CD148 WT, CS, or control GST proteins. As shown in **Figure 8B** (left panels), CD148 WT, but not CS or control GST, dephosphorylated p120 and β-catenin in a dose dependent manner, while its effects for E-cadherin were limited. p120 was more pronouncedly dephosphorylated by CD148 WT than was β-catenin. Further, as shown in the right panels, the dephosphorylation of p120 and β-catenin by CD148 WT was blocked by the addition of vanadate to the reaction. Collectively, these results demonstrate that both p120 and β-catenin, but not E-cadherin, serve as a substrate for CD148. It is of note that CD148 dephosphorylation of p120 Y228 residue was limited as compared with the overall p120 tyrosine dephosphorylation (**Figure S7**), suggesting that Y228 may not serve as a dephosphorylation site.

### p120 is required for the interaction of CD148 with E-cadherin/catenin complex

**Figure 9:**
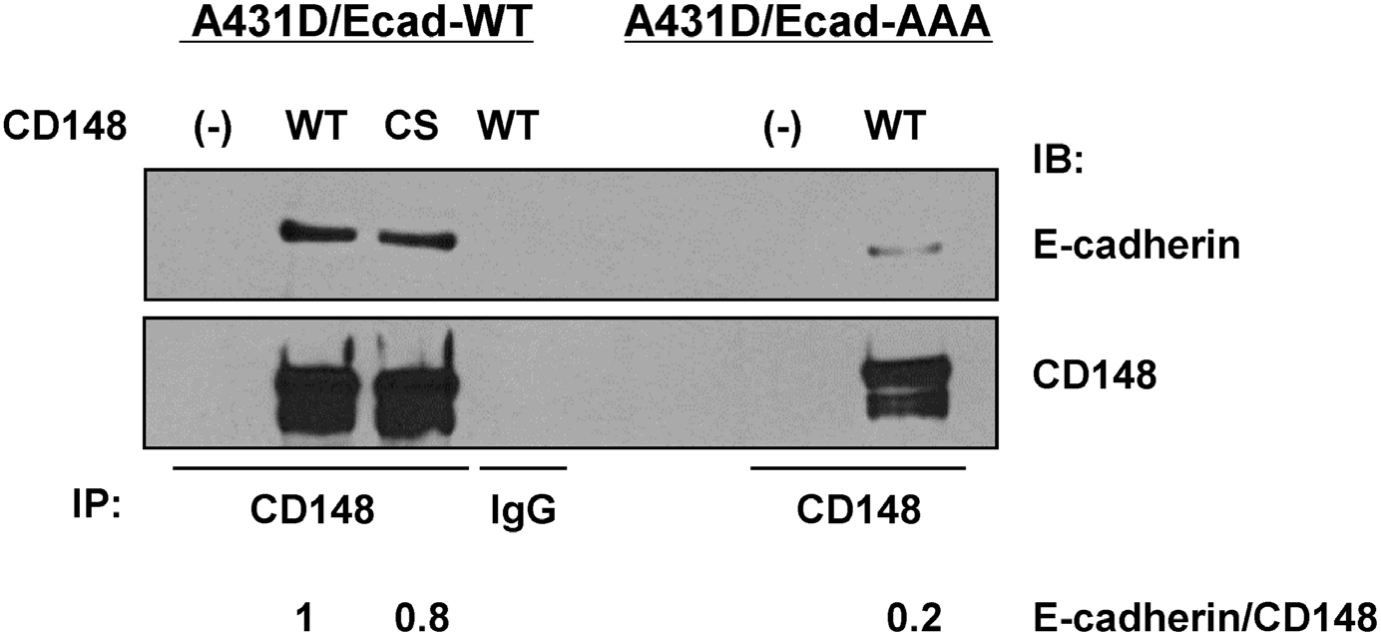
CD148 associates less with p120-uncoupled E-cadherin. CD148 was immunoprecipitated from A431D/E-cadherin WT or A431D/E-cadherin 764 AAA cells. Species-matched IgG was used as a control. The immunocomplexes were immunoblotted for E-cadherin and the amounts of CD148 were assessed by reprobing the membranes with anti-CD148. A ratio of E-cadherin to CD148 was quantified by densitometry. Data are representative of four independent experiments. Both CD148 WT and CS associate with wild-type E-cadherin, while CD148 association with p120-uncoupled E-cadherin is relatively limited.

A series of data has demonstrated that the association of E-cadherin with p120 is indispensable for the CD148 enhancement of E-cadherin cell adhesion. Further, *in vitro* assays (**Figure 8**) demonstrated that p120 serves as a substrate for CD148. These results suggest that p120-mediated signaling plays an essential role in this CD148 activity. However, it is also possible that the lack of p120 binding to E-cadherin disrupts the CD148-E-cadherin association as well as the CD148 signaling. Therefore, we addressed this issue by co-immunoprecipitation experiments. Shown in **Figure 9**, wild-type E-cadherin was co-immunoprecipitated with CD148 WT or CS in A431D/E-cadherin WT cells; however, the association of CD148 WT with p120-uncoupled E-cadherin 764AAA mutant was relatively limited in A431D/E-cadherin 764AAA cells, indicating that p120 is required for the association of CD148 with E-cadherin complex.

## Discussion

The present study demonstrates, for the first time, that: 1) CD148 promotes E-cadherin cell-cell adhesion concomitant with an increase in Rac1 activity; 2) CD148 reduces the tyrosine phosphorylation of p120 and β-catenin in E-cadherin contacts. Further, the *in vitro* data demonstrate that p120 and β-catenin serve as a substrate for CD148; 3) CD148 causes the dephosphorylation of the suppressive tyrosine residue in Src in E-cadherin contacts, increasing Src activity and possibly increasing the phosphorylation of p120 Y228 and Vav2 Y172; 4) p120 is required for the CD148 and E-cadherin association.

The CD148 enhancement of E-cadherin cell adhesion is accompanied by an increase in Rac1 activity. A body of evidence has shown a critical role of Rac1 in the establishment of cadherin adhesion. Rac 1 was shown to be recruited to the sites of E-cadherin contacts [51], and the introduction of dominant negative Rac1 was shown to block the formation of stable E-cadherin cell adhesion [52], [53]. Further, a recent study has shown that Rac1 recruitment and activation in cell-cell contact sites promotes the initiation, expansion, and consolidation of cadherin cell–cell adhesion [31]. Rac1 was also shown to mediate recruitment of actin to the clustered cadherin complexes and strengthen cadherin-mediated cell adhesion [51]–[55]. In addition, it was shown that Rac1 is required to maintain the mature cadherin adhesion [56]. Thus, an increase in Rac1 activity may be a pivotal event that explains the observed CD148 effects to expand E-cadherin contacts, generating more continuous and closely joined cadherin contacts, and to strengthen cell-cell adhesion.

**Figure 10:**
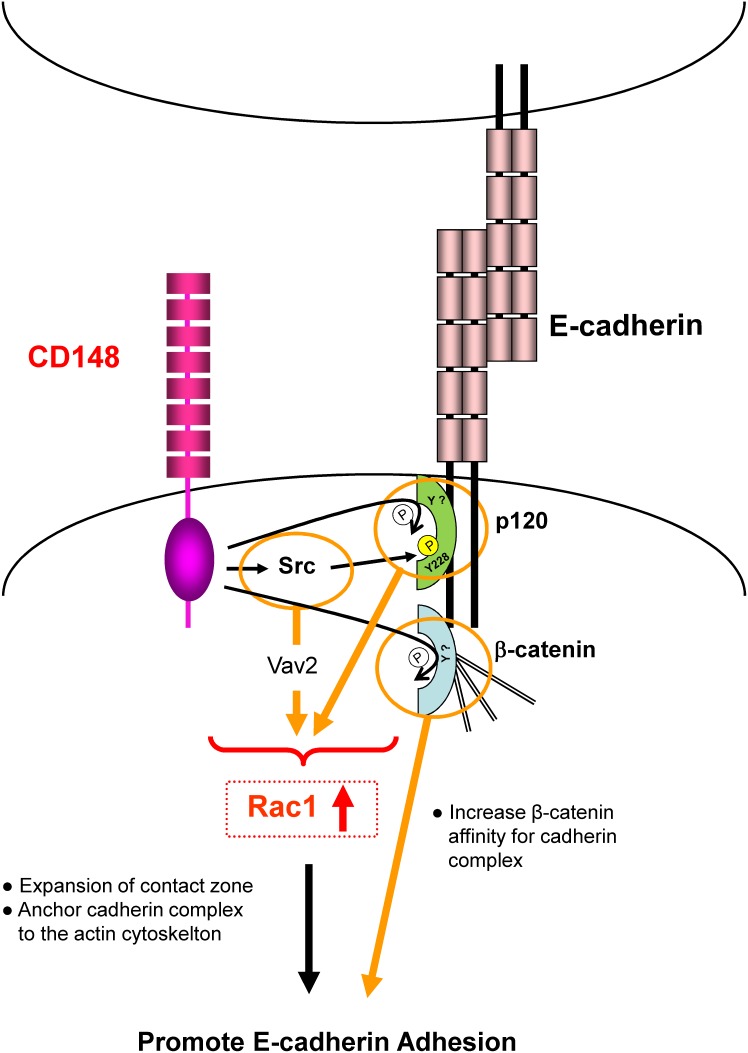
A Hypothetical model depicting CD148 regulation of E-cadherin cell-cell adhesion. CD148 dephosphorylates p120 and β-catenin in E-cadherin contacts. It also dephosphorylates the suppressive tyrosine residue (Y529) in Src, increasing Src activity and possibly enhancing the phosphorylation of Y228 in p120. These signaling events increase Rac1 activity and promote the expansion of contact zones and the stabilization of cadherin complexes, resulting in stronger E-cadherin cell-cell adhesion.

How does CD148 increase Rac1 activity in E-cadherin contacts? We hypothesize there may be three possibilities. First, it is well known that CD148 dephosphorylates the suppressive tyrosine residue in Src and increases its activity [19], [41], [42]. Further, Src was shown to activate Rac1 through the guanine exchange factor Vav2 and promote E-cadherin adhesion [43]. Therefore, CD148 could increase Rac1 activity through the Src-Vav2 pathway and facilitate E-cadherin cell adhesion. It is of note that CD148 dephosphorylation of Src is not observed in A431D/E-cadherin 764AAA cells; this finding suggests that the association of CD148 with E-cadherin complex may be required for the CD148 activation in cell-cell contacts. Src was also shown to increase Rac1 activity through the PI3 kinase pathway and promote E-cadherin adhesion [43], [57]; however, given the fact that CD148 dephosphorylates the p85 regulatory subunit and reduces PI3 kinase activity [18], this mechanism would be less contributory. Second, the lines of evidence suggest a role for p120 in regulating the Rac1 activity in E-cadherin contacts. The p120-uncoupled E-cadherin was shown to ablate the ability of E-cadherin to recruit and activate Rac1 and cause the defects in the formation of stable adhesive contacts [29]. This adhesive defect is rescued by introduction of constitutively active Rac1 [29]. Further, a recent study has shown that inhibition of the binding of VE-cadherin to p120 reduces adhesive contact area in a Rac1-dependent manner [30], suggesting that p120 expands the cadherin contact zone through Rac1. Cytoplasmic p120 was shown to interact with Vav2 and increase the Rac1 activity and cell motility [58], [59]. Further, recent studies have shown that the reduction of tyrosine phosphorylation of cytoplasmic p120 promotes its interaction with Vav2 and increases the Rac1 activity [59]. However, to date, the mechanism by which the cadherin-bound p120 regulates Rac1 activity is largely unknown. Also, the significance of tyrosine phosphorylation or dephosphorylation of the cadherin-bound p120 is poorly understood. In this context, it is of interest that Src-mediated tyrosine phosphorylation (Y217 and Y228) of p120 promotes the p120 and RhoA-GDP association, while Fyn-mediated tyrosine phosphorylation (Y112) inhibits the p120’s Rho guanine nucleotide dissociation inhibitor (RhoGDI) activity [60]. These findings suggest that tyrosine phosphorylation or dephosphorylation of the cadherin-bound p120 may regulate the p120 control of Rho family GTPases. Further investigation would be required to determine the significance of CD148 dephosphorylation and phosphorylation (Y228) of p120 in E-cadherin mediated Rac1 activation. Third, β-catenin is also dephosphorylated by CD148. A body of evidence has shown that tyrosine phosphorylation of β-catenin weakens cadherin function by dissociating β-catenin from cadherin or α catenin and that PTPs that dephosphorylate β-catenin enhance the cadherin adhesion [33]. However, there is at present no evidence that E-cadherin mediated Rac1 activation is regulated by the tyrosine phosphorylation or dephosphorylation of β-catenin. In this context, it is of note that Shp2-mediated tyrosine dephosphorylation of β-catenin promotes VE-cadherin cell adhesion in endothelial cells without altering Rho family GTPase activity [48]. Taken together, it is likely that CD148 dephosphorylation of β-catenin enhances the cadherin cell adhesion independent of Rho family GTPases. For this subject, we also assessed CD148 activity in dephosphorylating the Y654 tyrosine residue in β-catenin by an *in vitro* assay as the phosphorylation of this residue is known to reduce the affinity of β-catenin for cadherin [48]. However, prominent effects were not observed for this tyrosine residue (data not shown), suggesting that CD148 may dephosphorylate other tyrosine residues in β- catenin. Further investigation would be required to elucidate this possibility. A hypothetical model for CD148 regulation of E-cadherin cell-cell adhesion is shown in **Figure 10**.

Our data also demonstrate that p120 catenin is required for the association of CD148 with E-cadherin. Recent studies have shown that p120 prevents cadherin endocytosis and functions as a master regulator of cadherin stability and cell surface retention [24], [61]. Since p120-uncoupled E-cadherin is forcibly expressed in our cells, its expression levels are not notably reduced in this study. However, the increased internalization of p120-uncoupled E-cadherin may reduce its interaction with CD148. Alternatively, we noted that p120 significantly binds to the GST-CD148 WT protein as compared with GST *in vitro* (data not shown). This finding suggests that p120 may mediate the association of CD148 with E-cadherin as well as serve as a substrate.

In conclusion, the present study provides for the first time the functional and biochemical evidence about CD148 regulation of cadherin cell adhesion. Further investigation of this pathway should give a new insight into PTP regulation of cadherin function.

## Supporting Information

Figure S1 **Expression levels of the stably introduced CD148 are comparable to those in cultured human endothelial cells.** CD148WT-introduced or CD148-negative A431D/E-cadherin WT cells and human renal microvascular endothelial cells (HRMEC, J Am Soc Nephrol 10: 2135–2145, 1999) were stained with a PE-conjugated CD148 antibody (R&D System) and the expression levels of CD148 were assessed by flow cytometry (BD LSR II flow cytometer, BD Biosciences, San Jose, CA) as described previously (PNAS 109; 1985–1990, 2012).
(PDF)

Figure S2 **CD148 introduction promotes VE-cadherin contacts in HUVEC cells.** The recombinant adenovirus encoding HA-tagged CD148 WT or β-galactosidase (LacZ) were infected to subconfluent human umbilical vein endothelial cells (HUVEC, Lonza, Walkersville, MD) at a multiplicity of infection of 100 as described previously (PNAS 109; 1985–1990, 2012). At 48 h post infection, the cells were washed with PBS and fixed with 100% methanol (for VE-cadherin) or 2% paraformaldehyde followed by permeabilization with 0.02% saponin (for HA). The cells were immunostained with VE-cadherin (Cadherin 5, BD biosciences, San Jose, CA) or HA (mouse monoclonal, Covance, Princeton, NJ) antibodies followed by incubation with a secondary antibody (Alexa Flour 488 goat anti-mouse IgG, Invitrogen Corporation, Carlsbad, CA). The nucleus (purple) was counterstained with TO-PRO-3 reagent (Invitrogen, Carlsbad, CA). Cells were photographed with Zeiss LSM 510 confocal microscopy. CD148-overexpression expands VE-cadherin contacts, generating more continuous distribution, in HUVEC cells (upper panels). Anti-HA immunostaining indicates that HA-tagged CD148 is expressed in most of the cells (>90%) (lower panels).
(PDF)

Figure S3 **E-cadherin blocking antibody abolishes the CD148 effects to increase Rac1 activity in a calcium switch assay.** The CD148 effects increasing Rac1 activity were assessed by a calcium-switch assay in the presence (+) or absence (−) of an E-cadherin blocking antibody (1 µg/ml) (HECD1, Takara Bio, Madison, WI).
(PDF)

Figure S4 **Effects of CD148 in Rho-family GTPase activities in A431D cells.** CD148 WT-introduced or CD148-negative A431D cells were subjected to a hanging-drop assay. Rac1, Cdc42, and RhoA activities were measured at the indicated time points. The data show means ± SEM of quadruplicate determinations. In contrast to A431D/E-cadherin WT cells (Figure 5), an increase in Rac1 activity by CD148 WT is not observed in A431D cells.
(PDF)

Figure S5 **Comparison of p120, β-catenin, and Src tyrosine phosphorylation between A431D/E-cadherin WT and A431D/E-cadherin 764 AAA cells.** The tyrosine phosphorylation of p120, β-catenin and Src in E-cadherin contacts were compared between A431D/E-cadherin WT and A431D/E-cadherin 764 AAA cells (on the same gel) using a calcium-switch assay and immunoblot analysis. The membranes were reprobed with p120, β-catenin, and Src antibodies and a ratio of phosphorylated to total protein was quantified by densitometry.
(PDF)

Figure S6 **CD148 WT increases the tyrosine phosphorylation (Y172) of the membrane-associated Vav2 in E-cadherin contacts.** CD148WT-introduced or CD148-negative A431D/E-cadherin WT or A431D/E-cadherin 764AAA cells were subjected to a calcium switch assay. The cell membrane fraction was isolated using Qproteome Cell Compartment kit (QIAGEN, Valencia, CA) according to the manufacturer’s instruction. Vav2 was immunoprecipitated with anti-Vav2 (H-200, Santa Cruz Biotechnology, Santa Cruz, CA) and the phosphorylation of Vav2 was assessed by immunoblotting with a phospho (pY172)-Vav2 antibody (Santa Cruz Biotechnology, Santa Cruz, CA). The amounts of Vav2 were assessed by reprobing the membrane with anti-Vav2. Purity of the cell membrane fraction was assessed by anti-calnexin (H-70, Santa Cruz Biotechnology, Santa Cruz, CA) and anti-α tubulin (Vanderbilt Antibody and Protein Resource, Nashville, TN) immunoblotting. A ratio of phosphorylated to total Vav2 was quantified by densitometry (right panels). The data shows representative of four independent experiments. CD148 WT, but not CS, increases the phosphorylation of Vav2 in E-cadherin contacts in A431D/E-cadherin WT cells. This effect is not observed in A431D/E-cadherin 764 AAA cells.
(PDF)

Figure S7 **CD148 dephosphorylation of p120 Y228 residue is limited** ***in vitro*** **.** CD148 dephosphorylation of p120 Y228 was assessed *in vitro*, compared with the overall p120 tyrosine dephosphorylation. p120 was immunoprecipitated from the pervanadate (PV)-treated or untreated A431D/E-cadherin WT cells. The immunoprecipitates were incubated with GST or GST-CD148 proteins (1.0 µg) and its effects were assessed by immunoblotting using pY20 phosphotyrosine (pY) and p120 Y228 phospho-specific (Y228) antibodies. The amount of proteins was assessed by reprobing the membrane with a p120 antibody.
(PDF)
