# Supplementary figures and images for: CD148 Tyrosine Phosphatase Promotes Cadherin Cell Adhesion
Source: PLoS One. 2014 Nov 11;9(11):e112753. doi: 10.1371/journal.pone.0112753 (PMC4227875; doi:10.1371/journal.pone.0112753)

Takahashi K et al. Supplemental Figure S1

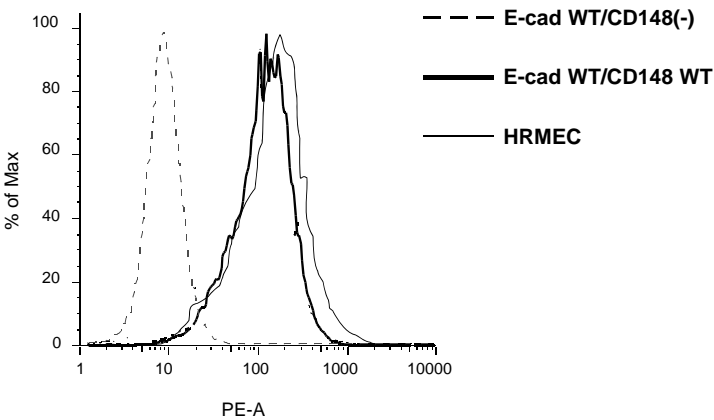

Supplement: Figure S1 — Expression levels of the stably introduced CD148 are comparable to those in cultured human endothelial cells. CD148WT-introduced or CD148-negative A431D/E-cadherin WT cells and human renal microvascular endothelial cells (HRMEC, J Am Soc Nephrol 10: 2135–2145, 1999) were stained with a PE-conjugated CD148 antibody (R&D System) and the expression levels of CD148 were assessed by flow cytometry (BD LSR II flow cytometer, BD Biosciences, San Jose, CA) as described previously (PNAS 109; 1985–1990, 2012). (PDF) [file pone.0112753.s001.pdf]

Takahashi K et al. Supplemental Figure S2

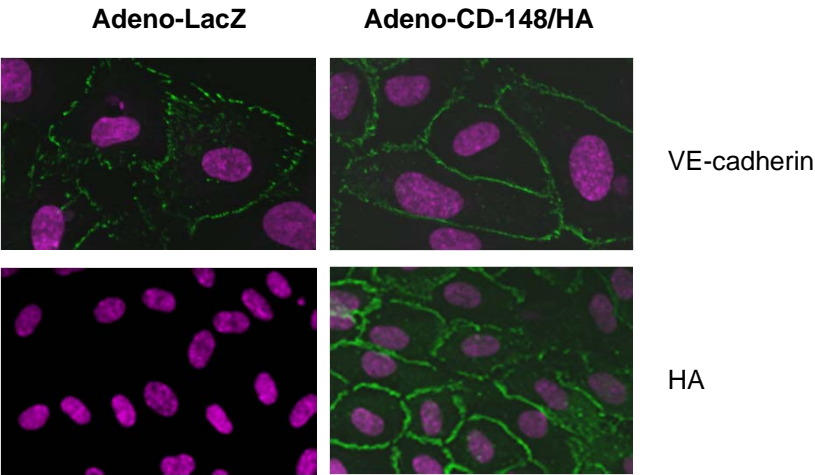

Supplement: Figure S2 — CD148 introduction promotes VE-cadherin contacts in HUVEC cells. The recombinant adenovirus encoding HA-tagged CD148 WT or β-galactosidase (LacZ) were infected to subconfluent human umbilical vein endothelial cells (HUVEC, Lonza, Walkersville, MD) at a multiplicity of infection of 100 as described previously (PNAS 109; 1985–1990, 2012). At 48 h post infection, the cells were washed with PBS and fixed with 100% methanol (for VE-cadherin) or 2% paraformaldehyde followed by permeabilization with 0.02% saponin (for HA). The cells were immunostained with VE-cadherin (Cadherin 5, BD biosciences, San Jose, CA) or HA (mouse monoclonal, Covance, Princeton, NJ) antibodies followed by incubation with a secondary antibody (Alexa Flour 488 goat anti-mouse IgG, Invitrogen Corporation, Carlsbad, CA). The nucleus (purple) was counterstained with TO-PRO-3 reagent (Invitrogen, Carlsbad, CA). Cells were photographed with Zeiss LSM 510 confocal microscopy. CD148-overexpression expands VE-cadherin contacts, generating more continuous distribution, in HUVEC cells (upper panels). Anti-HA immunostaining indicates that HA-tagged CD148 is expressed in most of the cells (>90%) (lower panels). (PDF) [file pone.0112753.s002.pdf]

Takahashi K et al. Supplemental Figure S3

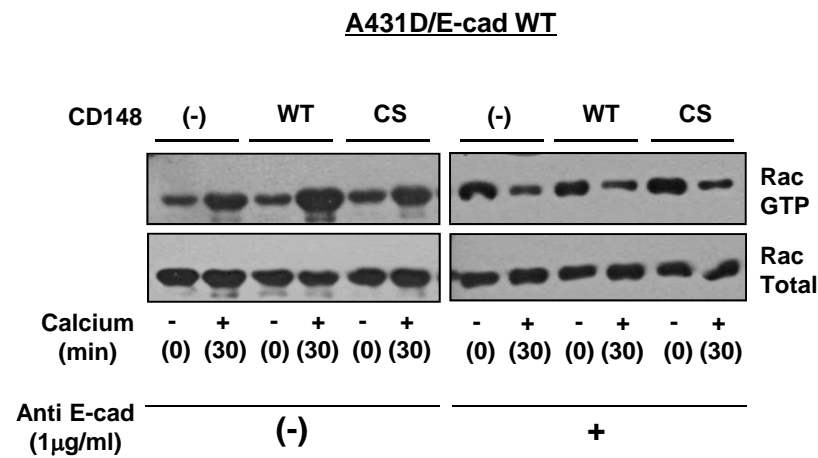

Supplement: Figure S3 — E-cadherin blocking antibody abolishes the CD148 effects to increase Rac1 activity in a calcium switch assay. The CD148 effects increasing Rac1 activity were assessed by a calcium-switch assay in the presence (+) or absence (−) of an E-cadherin blocking antibody (1 µg/ml) (HECD1, Takara Bio, Madison, WI). (PDF) [file pone.0112753.s003.pdf]

A431D Cells

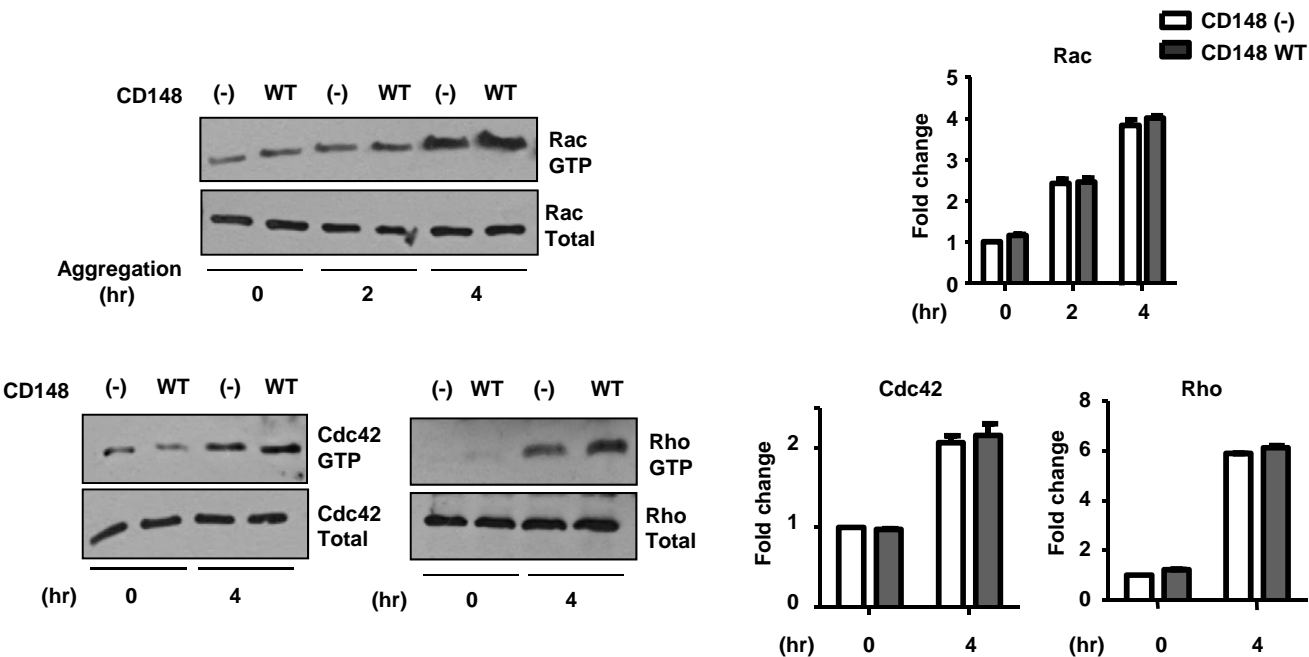

Supplement: Figure S4 — Effects of CD148 in Rho-family GTPase activities in A431D cells. CD148 WT-introduced or CD148-negative A431D cells were subjected to a hanging-drop assay. Rac1, Cdc42, and RhoA activities were measured at the indicated time points. The data show means ± SEM of quadruplicate determinations. In contrast to A431D/E-cadherin WT cells (Figure 5), an increase in Rac1 activity by CD148 WT is not observed in A431D cells. (PDF) [file pone.0112753.s004.pdf]

Takahashi K et al. Supplemental Figure S5

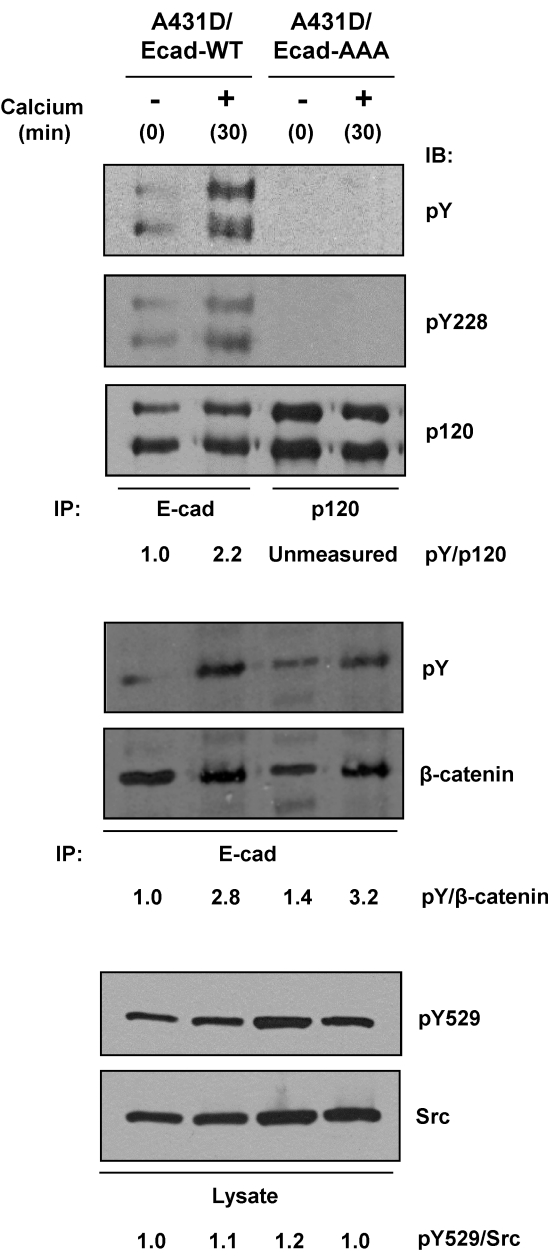

Supplement: Figure S5 — Comparison of p120, β-catenin, and Src tyrosine phosphorylation between A431D/E-cadherin WT and A431D/E-cadherin 764 AAA cells. The tyrosine phosphorylation of p120, β-catenin and Src in E-cadherin contacts were compared between A431D/E-cadherin WT and A431D/E-cadherin 764 AAA cells (on the same gel) using a calcium-switch assay and immunoblot analysis. The membranes were reprobed with p120, β-catenin, and Src antibodies and a ratio of phosphorylated to total protein was quantified by densitometry. (PDF) [file pone.0112753.s005.pdf]

Takahashi K et al. Supplemental Figure S6

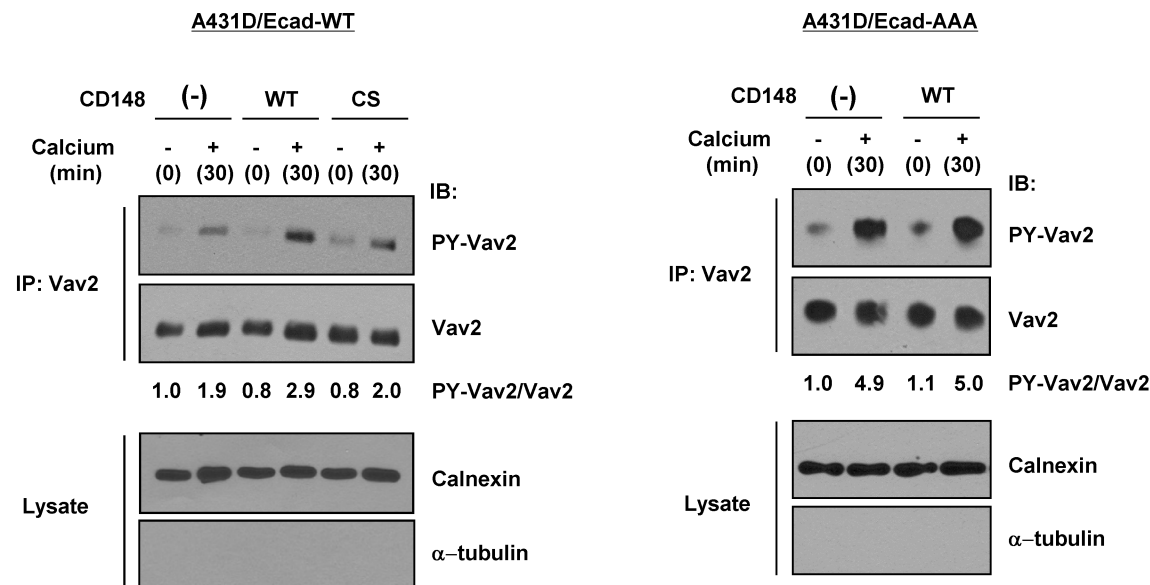

Supplement: Figure S6 — CD148 WT increases the tyrosine phosphorylation (Y172) of the membrane-associated Vav2 in E-cadherin contacts. CD148WT-introduced or CD148-negative A431D/E-cadherin WT or A431D/E-cadherin 764AAA cells were subjected to a calcium switch assay. The cell membrane fraction was isolated using Qproteome Cell Compartment kit (QIAGEN, Valencia, CA) according to the manufacturer’s instruction. Vav2 was immunoprecipitated with anti-Vav2 (H-200, Santa Cruz Biotechnology, Santa Cruz, CA) and the phosphorylation of Vav2 was assessed by immunoblotting with a phospho (pY172)-Vav2 antibody (Santa Cruz Biotechnology, Santa Cruz, CA). The amounts of Vav2 were assessed by reprobing the membrane with anti-Vav2. Purity of the cell membrane fraction was assessed by anti-calnexin (H-70, Santa Cruz Biotechnology, Santa Cruz, CA) and anti-α tubulin (Vanderbilt Antibody and Protein Resource, Nashville, TN) immunoblotting. A ratio of phosphorylated to total Vav2 was quantified by densitometry (right panels). The data shows representative of four independent experiments. CD148 WT, but not CS, increases the phosphorylation of Vav2 in E-cadherin contacts in A431D/E-cadherin WT cells. This effect is not observed in A431D/E-cadherin 764 AAA cells. (PDF) [file pone.0112753.s006.pdf]

Takahashi K et al. Supplemental Figure S7

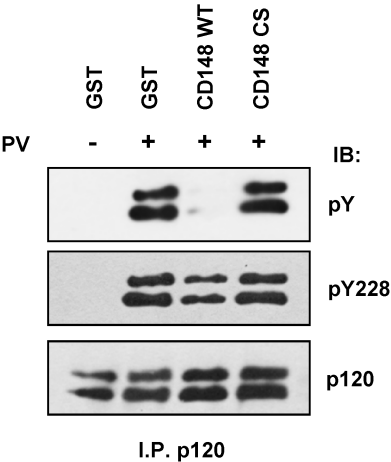

Supplement: Figure S7 — CD148 dephosphorylation of p120 Y228 residue is limited in vitro . CD148 dephosphorylation of p120 Y228 was assessed in vitro, compared with the overall p120 tyrosine dephosphorylation. p120 was immunoprecipitated from the pervanadate (PV)-treated or untreated A431D/E-cadherin WT cells. The immunoprecipitates were incubated with GST or GST-CD148 proteins (1.0 µg) and its effects were assessed by immunoblotting using pY20 phosphotyrosine (pY) and p120 Y228 phospho-specific (Y228) antibodies. The amount of proteins was assessed by reprobing the membrane with a p120 antibody. (PDF) [file pone.0112753.s007.pdf]
